# Smartphones and Wearables as a Method for Understanding Symptom Mechanisms

**DOI:** 10.3389/fpsyt.2019.00949

**Published:** 2020-01-17

**Authors:** Benjamin Griffin, Kate E. A. Saunders

**Affiliations:** ^1^Wadham College Oxford, Oxford, United Kingdom; ^2^Department of Psychiatry, Oxford University, Warneford Hospital, Oxford, United Kingdom; ^3^Oxford Health NHS Foundation Trust, Warneford Hospital, Oxford, United Kingdom

**Keywords:** wearables, symptom monitoring, mechanism, psychological treatments, digital technology, mental health

## Abstract

While psychological treatments have been shown to be effective in treating psychiatric disorders, the mechanism of their therapeutic effect is less well understood. An improved mechanistic understanding of psychiatric disorders and their treatments would enable refinement of existing interventions, and more targeted intervention and the development of new treatments. A major limitation in understanding the mechanism of effect in psychological treatments has been the challenge of capturing what happens outside of the clinical setting. The development of new digital technologies such as smartphones and wearables enables much more inter-session data to be collected. The rapid evolution of smartphones and wearable technologies, combined with the ubiquity of mobile networks means that is possible for patients to provide regular, longitudinal, and high-resolution data. This allows a previously inaccessible and untapped stream of a specific patient’s behaviours, moods, activities, and thoughts to be quantified. Monitoring through such technologies may be of therapeutic value, improving self-awareness and promoting mentalization. Smartphones and wearable technologies can also be used to delivered therapies remotely. Digital technologies enable new insights to be gained into the lived experience of mental disorder enabling current treatments to be refined and personalised, as well as generating new targets for future treatment development. In this article we discuss how such technologies are improving our understanding of psychiatric disorder, informing psychological treatments before considering the future potential of such technologies. We will also consider the challenges and ethical concerns of such approaches.

## Introduction

Direct clinical evidence fairly incontrovertibly demonstrates that psychological treatments are effective for the vast majority of mental health disorders, including mood (1), anxiety (2), eating (3), psychotic (4), and personality disorders (5). The diversity of such psychological approaches in treating mental conditions is vast: spanning from Freud’s ‘psychoanalytical theory’, in which emphasis is placed upon exploring the unconscious mind and how an individual’s childhood experiences may have shaped this, to cognitive behavioural therapies (CBT), which aim to be more problem-focused and action-oriented.

However, despite these differences in the way mental health problems are conceptualised, construed, and thus treated by the different schools of psychological therapies, evidence suggests that most psychological therapies have some effectiveness in treating mental disorder. It has previously being claimed that all psychological therapies are equal (6, 7), yet a number of meta-analyses suggest that CBT may be more effective than other forms of psychological therapies (8). However, CBT’s superiority does not extend to all psychiatric conditions.

This lack of clarity has led to the hypothesis that psychological treatments all work *via* “non-specific” mechanisms, an argument given more weight by the fact that the precise mechanisms by which therapies work are largely unknown. Some commentators have suggested that this “non-specific mechanism” may be better characterised as the “therapeutic alliance” between patient and practitioner, as this is one of the most consistent predictors of psychotherapy outcome (7, 9). Proponents of this theory argue that the relationship between therapist and patient is more important that the specific school of psychotherapy used, and highlight the difficulty in controlling for this in clinical trials.

Our ability to assert which therapy is most effective for a given condition is also limited by our ability to measure improvement. The highly complex and dynamic lived experience of an individual with a mental illness is often reduced to what can be easily coded and analysed in studies: crude scoring systems which provide a snapshot of symptomology on a single day, rather than a longitudinal view of function over weeks or months. Psychiatric conditions are highly heterogenous both in their aetiology and their presentation and the optimal treatment for one individual may differ significantly to that for another. However, the majority of trials present between group differences and are rarely powered to explore individual predictors of treatment response.

Recent technological advances provide investigators with a new set of tools which enable real-time monitoring and the addition of personalised outcome measures. A number of preliminary studies have demonstrated the immense power of these technologies, allowing a previously inaccessible and untapped stream of a specific patient’s behaviours, moods, activities, and thoughts to be measured.

## A Revolution in Data Collection

Digital technologies enable researchers to collect and analyse vast amount of naturalistic data i.e. data collected from individuals in their natural environment (10). This approach, sometimes referred to as ‘digital phenotyping’ (11), is defined as moment-by-moment quantification of the individual-level human phenotype in-situ using data from personal digital devices (12). Previously, data collection and treatment monitoring has been limited to being carried out in person in clinical settings, thus providing data sets of limited size and utility.

Such digital approaches provides a solution to the inherent limitations of cross-sectional self-reported data collected in a clinical setting by providing instantaneous and more objective sources of measurement. This can be utilised in the context of psychological therapies to better inform and personalise treatment, explore mechanisms, treatment concordance and provide more accurate quantification of treatment outcome.

## Symptom Monitoring

Symptom monitoring forms part of a number of therapies but the momentary nature of smartphone-based monitoring enables more detailed inter-session information to be collected, leading to better informed therapy sessions and provide more specific information about when in therapy positive changes begin to occur. Repeated sampling of behaviours, experiences and feeling in real time, termed ecological momentary assessment (EMA) (13) can provide insight into the temporal relationship of symptoms which may then inform subsequent therapy. The utility of EMA has already begun to be realised across a range of studies, in a variety of mental disorders. For example, prospective monitoring of individuals with post-traumatic stress disorder (PTSD) revealed that PTSD symptoms predicted subsequent anger issues, but the inverse is not true suggesting that trauma may be highly relevant to anger management (14). In panic disorder EMA has revealed the nature of the relationship between anticipatory anxiety and panic attacks supporting the underlying cognitive theories in extinction research (15). These findings have clear therapeutic implications. In bipolar disorder mood monitoring has revealed that mood instability is a far more prominent feature than previously thought (16), acting as a prodromal feature, a marker of severity and has an impact upon inter episode functioning. This not only has potential implications for the diagnostic criteria, in which mood symptoms are categorised into discreet episodes, but also how psychotherapies may be best designed to treat such patients.

## Passive Data Collection

While rating symptom scores throughout the day are extremely valuable it relies on individuals completing their ratings and assumes their self-appraisal to be accurate. It has been long established that symptom reporting can be a therapeutic intervention in itself, but patients can become fatigued with answering the same questions on a regular basis and there are concerns that active monitoring can act as a reminder of illness. The temporal resolution is inherently limited to the frequency of prompts a patient is prepared to tolerate. In addition when people become unwell often stop reporting their symptoms or do so in a less reliable manner making the interpretation of the data challenging. One of the significant advantages of new smartphone and wearable technologies is their ability to collect passive data streams. Passive data is information that is collected automatically without the participant having to do anything. Passive monitoring can take many forms, with one of the most promising of these being the ability to quantify human-computer interactions. This not limited to measuring just *what* is being done, but rather *where, when*, and *how* it is being done, for example how we interact with mobile phones. A recent small-scale study demonstrated subtle aspects of typing and scrolling such as the latency between space and character or the interval between scroll and click can be used as surrogates for affective states and cognitive traits, (17). Aspects of phone use such as length and number of outgoing phone calls, the number of outgoing text messages per day (18), the variability in geolocation have all been shown to correlate with severity of mood symptoms (19).

Passive data is continuous, often multimodal in nature and inherently ecologically valid while minimising the burden on patients. In the context of psychological treatments this technology provides a platform for testing different psychological theories. For example theories that relate to interpersonal relationships or emotional dynamics often require much greater temporal resolution that self-report is able to provide. Koval and Kuppens (20) have proposed the theory of emotional inertia where the autocorrelation of an individual’s emotions over time predict depressive symptoms. The use of continuous, high frequency passive data allows such theories to be tested outside of laboratory settings. In therapies that have significant behavioural components passive data collection can not only measure compliance but also the temporal relationships between behavioural and other symptoms.

Passive data may also enable treatments to be more accurately targeted both temporally and situationally. Such digital approaches raise the possibility of technologically informed individualisation. Through analysing the data stream of one patient, it may be possible to algorithmically predict future events based on the past: for example, in bipolar disorder where the notion of idiosyncratic ‘relapse signatures’ has begun to be characterised (21), it is conceivable that such prodromal features could be detected digitally. Moreover, these insights not only inform when intervention may best be delivered, but act as a psychoeducation tool enabling patients to develop a better understanding of their disorder. Digital CBT interventions such as Sleepio are already integrating data streamed from wearable devices into their interventions thus providing intervention which is based this objectively collected data.

Passive data collection has also extended to the use of optical sensors mounted in homes or clinical settings. These sensors can detect patient’s location, activities, and vital signs (heart rate and respiratory rate) and have been demonstrated to be well tolerated by patients (22). Such digital observational systems are currently confined to inpatient environments but could be used in a range of clinical or home settings to monitor a range of patient behaviours including real-time measurement of group process and interpersonal dynamics.

## Challenges and Controversies

While such technologies clearly represent a huge opportunity they also present complex practical and ethical issues to those working in this area.

New technologies undoubtedly allow us to collect data in much greater quantities, at higher temporal resolution across multiple different modalities. There are concerns that data collection in this area is often not ‘hypothesis driven’: large amounts of data are collected over a long amount of time without researchers having anything specific to test. Vast stores of data may accumulate over many years before analysis and subsequent use might occur. The ways in which data may be used in the future appear almost infinite: this presents a problem in the fact it is not possible to consent those using such technologies currently as to what their data may be used for in time to come. Such large volumes of data present a huge analytic challenge as for any of these technological advances to be realised the data they provide must be valid, interpretable and reliable. Missing data is a common challenge especially in self-reported data. Data may be missing for multiple technical (for example power cuts, battery failure) practical or clinical reasons. This can pose a challenge when analysing the data as the methods for dealing with missing data may have a significant impact upon the findings. If data is missing at random and it may seem reasonable to use imputation (the process of replacing missing data with a substituted value), but if the missing data conveys information about a clinical state (or both), any attempt to impute data may in fact lead to the loss of data. Knowing which of these respective approaches is correct for any given data set is almost impossible.

Analysing large complex data sets often requires techniques that cross traditional disciplinary divides drawing on approaches from engineering, maths and computer science. These analytic approaches are key to the viability of using digital data to provide meaningful within subject outputs. Such approaches are generally not well understood by clinical staff or easily interpretable and are likely to require close collaboration and changes to training of clinical staff.

The regular use of smartphones or other wearable devices also requires patients to keep devices charged and turned on. They need to be able to access network connections. Sensor data uses significant amounts of battery life which may lead to data collection being switched off. Sensors differ significantly between devices and operating systems making the accurate quantification and comparison of data between individuals challenging.

There are risks surrounding data security and patient confidentiality. This is well exemplified by research carried out by Nicholas et al., reporting on mobile apps for bipolar disorder: none of the symptom apps identified by the group had been subject to rigorous research or cited published material, and only 22% had a privacy policy (23). For apps and wearable devices to be acceptable to patients there needs to be clear and rigorous mechanisms to uphold privacy. Efforts are now being made to mitigate such risks: the Department of Health and Social Care has recently published a code of conduct for data-driven health and care technology, outlining 10 key principles for safe and effective digital innovations, and detailing 5 government commitments aiming to ensure that the health care system is ready and able to safely adopt innovations in technology on a large scale. The Medicines and Healthcare products Regulatory Agency (MHRA) has recently issued updated guidance to help identify whether software and health apps are medical devices and the National Institute for Health Research (NIHR) is now offering regulatory advice for those seeking to develop novel medical technologies.

The acceptability of digital data collection to patients is central to the uptake of such approaches in research and clinical settings. A number studies, including a multi-site collaborative study which involved patients with a range of disorders, suggest that the use of digital technologies is highly acceptable to patients, and levels of compliance appear to relatively high (24, 25) . However, concerns have been raised regarding the propensity for individuals to develop preoccupation and paranoia with their continual monitoring (26). A large follow-up study of a cohort of bipolar patients found that 20% of those offered online mood monitoring declined citing concerns that longitudinal monitoring could adversely affect mood (27).

These somewhat disparate findings may be explained by the fact that the studies in which use of digital technologies was more positively appraised patients received face to face training from the investigators, compared to the use of post and email to train patients in the other studies. This suggests that in order to maximise the acceptability of digital technologies to patients, efforts should be made to optimise levels of training and support offered to them.

There is evidence linking increased use of smartphones to decreased psychological wellbeing in adolescents. When controlling for other factors, researchers found that increased mobile phone use was linked to subsequent decline in well-being, while those adolescents spending the least amount of time on electronic communications reported higher levels of well-being (28). This ties into research indicating that remote monitoring may increase symptoms of anxiety and mental distress. While these studies are far from conclusive, and only considered very specific cohorts so may not be more widely applicable to psychiatric patients, it is reasonable to question whether encouraging patients to spend more time on their mobile phones may do more harm than good in some cases.

## Conclusion

The use of remote monitoring using digital technologies in psychiatry has expanded exponentially in recent years, providing researchers and clinicians with a hugely powerful and diverse set of new tools to explore and treat psychiatric disease. Unprecedented quantities and types of data from naturalistic settings promises to revolutionise our understanding of symptoms in this currently poorly understood area of medicine. This will enable the more rigorous scrutiny of the mechanism of action of existing psychological treatments as well as the generation of new psychological treatment targets. Whilst the impact of digital technologies is likely to be positive, a number of ethical and practical issues have to be considered. Actively seeking to involve patients in the development of such technologies, and providing adequate training and support for those using the devices and apps is likely to be key to the success of digital approaches. Greater effort should be made to understand the wider implications of the use of such technologies in psychiatry, with the aim of balancing the clinical and research possibilities against the health of patients, and the safety of their data.

## Author Contributions

KS and BG jointly co-authored the manuscript.

## Funding

KS is supported by the National Institute for Health Research (NIHR) Oxford Health Biomedical Research Centre.

## Conflict of Interest

KS is the digital phenotyping lead for the NIHR Oxford Health Biomedical Research Centre.

The remaining author declares that the research was conducted in the absence of any commercial or financial relationships that could be construed as a potential conflict of interest.
